# Socialization, legitimation and the transfer of biomedical knowledge to low- and middle-income countries: analyzing the case of emergency medicine in India

**DOI:** 10.1186/s12939-018-0824-y

**Published:** 2018-09-24

**Authors:** Veena Sriram, Asha George, Rama Baru, Sara Bennett

**Affiliations:** 1 Center for Health and the Social Sciences, University of Chicago, 5841 S. Maryland Avenue, MC 1005, Suite M200, Chicago, IL USA; 2 School of Public Health, University of the Western Cape, Robert Sobukwe Road, Bellville, 7535 Republic of South Africa; 3 Centre of Social Medicine and Community Health, School of Social Sciences, Jawaharlal Nehru University, New Mehrauli Road, Munirka, New Delhi, 110067 India; 4 Department of International Health, Johns Hopkins Bloomberg School of Public Health, 615 N Wolfe St, Baltimore, MD 21205 USA

**Keywords:** Medical specialization, Power, India, Transnational, Diaspora, Health systems, Emergency medicine, Emergency care, Knowledge transfer

## Abstract

**Background:**

Medical specialization is a key feature of biomedicine, and is a growing, but weakly understood aspect of health systems in many low- and middle-income countries (LMICs), including India. Emergency medicine is an example of a medical specialty that has been promoted in India by several high-income country stakeholders, including the Indian diaspora, through transnational and institutional partnerships. Despite the rapid evolution of emergency medicine in comparison to other specialties, this specialty has seen fragmentation in the stakeholder network and divergent training and policy objectives. Few empirical studies have examined the influence of stakeholders from high-income countries broadly, or of diasporas specifically, in transferring knowledge of medical specialization to LMICs. Using the concepts of socialization and legitimation, our goal is to examine the transfer of medical knowledge from high-income countries to LMICs through domestic, diasporic and foreign stakeholders, and the perceived impact of this knowledge on shaping health priorities in India.

**Methods:**

This analysis was conducted as part of a broader study on the development of emergency medicine in India. We designed a qualitative case study focused on the early 1990s until 2015, analyzing data from in-depth interviewing (*n* = 87), document review (*n* = 248), and non-participant observation of conferences and meetings (*n* = 6).

**Results:**

From the early 1990s, domestic stakeholders with exposure to emergency medicine in high-income countries began to establish Emergency Departments and initiate specialist training in the field. Their efforts were amplified by the active legitimation of emergency medicine by diasporic and foreign stakeholders, who formed transnational partnerships with domestic stakeholders and organized conferences, training programs and other activities to promote the field in India. However, despite a broad commitment to expanding specialist training, the network of domestic, diasporic and foreign stakeholders was highly fragmented, resulting in myriad unstandardized postgraduate training programs and duplicative policy agendas. Further, the focus in this time period was largely on training specialists, resulting in more emphasis on a medicalized, tertiary-level form of care.

**Conclusions:**

This analysis reveals the complexities of the roles and dynamics of domestic, diasporic and foreign stakeholders in the evolution of emergency medicine in India. More research and critical analyses are required to explore the transfer of medical knowledge, such as other medical specialties, models of clinical care, and medical technologies, from high-income countries to India.

## Background

The globalization of biomedicine has spread ‘western-style’ health care around the world, driven by a host of contextual factors that include colonialism and post-colonial aspirations related to modernism, science and technology and the commodification of health care [1–5]. Medical specialization is a central feature of biomedicine, and is a growing aspect of health systems in many low- and middle-income countries (LMICs) [6]. Specialization arguably deepens knowledge, ideas and technology related to health, and could contribute to improved health outcomes [7]. However, it could also contribute to a disproportionate focus on hospital-based specialized care, often at the expense of primary care or public health approaches that may benefit marginalized populations [6, 8, 9].

Within the context of biomedicine in India, medical specialization strongly influences service delivery, financing and workforce distribution. India is considered a ‘mixed health system’, consisting of an expansive public sector and an even broader, heterogeneous private sector [10]. Since the mid-1980s, the for-profit private sector has significantly invested in tertiary hospitals in primarily urban settings, steadily increasing its share of hospitals and beds in the country [11–13]. These tertiary care hospitals primarily focus on specialist and super-specialist care, some driven by India’s booming medical tourism industry [14–16]. Furthermore, policies to expand centrally-supported public sector tertiary hospitals and medical colleges in all states have received attention from national political parties [17, 18].

While tertiary care remains a small percentage of the total numbers of beds and institutions in the public and private sectors [19], its rising share fundamentally alters decision-making on the part of both student doctors and patients. Studies have found that the vast majority of Indian medical students wish to specialize after their undergraduate medical education [20–22], a trend amplified by the rise of private medical education and associated escalating costs and investments in specialty hospitals, largely in the private sector [15]. Finally, research has also shown that patients in India, including those utilizing the public sector, exhibit a strong preference for specialist doctors [23].

In many LMICs, including India, stakeholders from high-income countries actively promote the uptake and integration of new medical specialties in the health system – through training in high-income settings, ‘mission’ medical visits for foreign doctors in LMICs, establishment of medical colleges and training institutes and the diffusion of healthcare technology [3, 24, 25]. These stakeholders can broadly be categorized in two groups – the diaspora, defined as “emigrants and their descendants, who live outside the country of their birth or ancestry, either on a temporary or permanent basis, yet still maintain affective and material ties to their countries of origin” [26], and those who are non-diasporic, or ‘foreign’ to those countries. The increase in physician migration from LMICs to primarily high-income countries in the last several decades due to a host of personal, political, societal and systemic issues [27] has significantly amplified the role of the diaspora in promoting biomedicine in their home countries [27–32], a phenomenon that has been characterized as ‘brain circulation’ [32]. This phenomenon does not occur in a unidirectional, homogenous way; the diaspora of any country or region consists of multiple sub-groups – such as recent emigrants and first-, second- or third-generation high-income country citizens – reflecting a mosaic of ideologies, identities, and goals [33]. Finally, the diaspora may play a role in brokering broader transnational networks and partnerships, defined as “sustained ties of persons, networks and organizations across the borders of multiple nation-states, ranging from weakly to strongly institutionalized forms.” [34].

The involvement of Indians returning from professional experience in the health sectors of high-income countries, as well as diasporic physicians, has been a major theme in the development of the Indian health system, with increasing contemporary salience [33, 35, 36]. Such involvement is reflective of the vast array of political, cultural, religious, economic, scientific and technological interactions between the vast, global Indian diaspora and domestic stakeholders in India [33, 37]. Focusing specifically on biomedical, hospital-based care, the past several decades have seen an uptick in the involvement of these actors. These Indian stakeholders have forged transnational partnerships by linking medical institutions in their ‘home’ countries with medical institutions in high-income countries where they have worked or completed training prior to returning to India [24]. Diasporic physicians have permanently returned to India establish health facilities modeled along the lines of systems in high-income settings, most notably, Apollo Hospitals in 1983 [36, 38]. Diaspora-led associations such as the American Association of Physicians of Indian Origin actively facilitate the transfer of knowledge and technology to India through educational visits and conferences, financial and in-kind donations, and professional and scientific partnerships [38–41]. Such diaspora-driven partnerships have been encouraged by state and central governments in India as early as the mid-1980s, increasingly in the context of syncing with India’s neo-liberal economic reforms [37, 42, 43]. Finally, numerous academic medical institutions in high-income countries have established partnerships with Indian medical institutions focusing on a wide range of health issues [44].^1^

These types of activities, interventions and partnerships abound with issues related to power – around the positionality of those individuals and institutions seeking to make an impact in India, their relationships with the Indian medical community and with Indian patients. Yet few have critically examined the role of the diaspora and transnational institutional partnerships in hospital-based biomedical care in India, a significant gap in the literature given the increasingly important role of these stakeholders in shaping health policy and systems in India [45]. Such analyses would interrogate the largely positive narrative in the development literature around transnational flows of ‘knowledge, ideas and know-how’ from high-income countries to LMICs, in which the diaspora are important conduits [45].

The recent development of emergency medicine in India is an opportunity to critically examine the transfer of knowledge from high-income countries to LMICs within the context of transnational flows brokered by diasporic ties, and to understand the role of diasporic and foreign stakeholders in shaping health policy and systems in LMIC contexts. Emergency medicine is a relatively recent medical specialty in India that has been actively promoted by several foreign and diasporic stakeholders through transnational partnerships since the 1990s. These stakeholders included diaspora-led professional associations, international professional associations, several medical institutions and individuals working in a personal capacity. The presence of emergency medicine as a specialty has seemingly contributed to improvements in emergency care in India, such as the increased availability of organized Emergency Departments, protocols for service delivery, and short- and long-term training programs for health workers [46]. However, the evolution of emergency medicine, rapid in comparison to other medical specialties, has also seen fragmentation amongst key stakeholders, disagreements about the overarching policy objectives, and a lack of standardization in curricula and protocols [47]. The role of stakeholders from high-income countries in the development of emergency medicine has been previously written about in descriptive terms [48, 49], but has not been subject to rigorous research and critical analysis.

These issues were the subject of a doctoral dissertation undertaken by the first author, where the research focus was the development of new medical specialties in India through an examination of the recent evolution of emergency medicine. Specifically, we investigated three phases of the policy cycle – political prioritization [50], policy formulation, and implementation. We also contextualized the policy process by analyzing the regulatory landscape in India for recognition and training for new medical specialties [51]. The objective of this paper is to draw upon the development of emergency medicine in India to examine the role of high-income country stakeholders in the transfer of medical knowledge to India through the lens of two interlinked concepts in the field of power – socialization and legitimation – enabling us to understand the building of value commitments towards a particular idea. Analyzing power in this case allows us to investigate both the influence of ideas emerging from high-income countries, and the involvement of foreign and diasporic stakeholders in actively promoting pathways of medical specialization in an LMIC setting. By applying these two concepts, we seek to understand the visible and hidden forms of power that shape and influence health policy and systems in contexts such as India. Medical specialization is also an issue that largely sits at the margins of health policy and health systems, but one that continues to reshape and redefine health systems in many LMICs [9, 52]. Our intent here is not to determine whether the introduction of the specialty displaced other national priorities, as it is beyond the scope of this paper to assess the relative impact of the specialty in the policy agenda. Rather, our goal is to examine the transfer of medical knowledge from high-income countries to LMICs with a particular focus on diasporic and foreign stakeholders and the means that they have to build societal support and commitment to particular ideas, and in turn how this shapes health priorities in India.

### Conceptual background

Power dynamics shape health systems around the world, and the study of power has emerged as an important, yet underexplored, theme in health policy and systems research in LMICs [53–55]. Scott [56] discusses two broad elementary forms of power – corrective influence and persuasive influence, where corrective influence works through force and manipulation to achieve outcomes, while persuasive influence works through ‘the offering and acceptance of reasons for acting in one way rather than another’. The concepts of socialization and legitimation represent persuasive forms of power that facilitates the building of norms to a particular idea [56]. The arguments used to advance a particular cause might be seen as ‘especially compelling because of their particular character or competence’. Socialization and legitimation, representing both passive and active forms of value building, may overlap given the role of elite epistemic communities in policymaking [57, 58].

Socialization explains the voluntary adoption of an idea or a course of action, due to a perception of that idea being superior, modern or advanced [58]. For example, a particular health policy might be more attractive due to its origination and utilization in a high-income setting, or through the promotion of social norms pertaining to the policy by powerful international actors [59]. Stakeholders also sometimes use ‘inferential shortcuts’ in assessing the suitability of a policy or intervention to their setting, and often limit adaptation due to anchoring, whereby actors ‘confine modifications to peripheral aspects and retain the innovation’s design principles’ [60].

Legitimation refers to processes by which an idea or course of action is given legitimacy by building supportive societal norms and commitment [61, 62]. In contrast to socialization, where ideas are more passively diffused, legitimation suggests more active involvement of those promoting the idea in building value commitments toward it [56]. Legitimacy is inherently subjective, produced through an interaction between actors, institution and context. Several forms of legitimacy have been put forward, including output legitimacy, substantive legitimacy, and procedural legitimacy [63, 64]. In health policy, legitimation may occur through processes such as building community, network and/or societal commitment to an idea or policy [65], the role of medical expertise in generating support for population health challenges, the evolution of political priority amidst stakeholder pluralism and weak governance structures [63], and the transition from indigenous knowledge to professionalization and regulation [66].

Given the focus of this study, it is useful to reflect on the sources of power underlying domestic, diasporic and foreign stakeholders engaged in the socialization and legitimation of medical specialties in India. Domestic stakeholders, specifically those who have resettled in India following professional experience in high-income countries, may be perceived as having superior qualifications and knowledge when compared to domestic stakeholders whose training and experiences have been exclusively based in India. These returned Indians may also draw upon regional, caste, educational or political networks to build cultural capital that can be used to advance initiatives and partnerships [38]. Diasporic stakeholders who have settled in high-income countries may similarly be perceived as having superior technical knowledge, and in addition to their existing regional, caste, educational or political connections, may also draw upon strong financial support and elite standings [67], Finally, foreign stakeholders may be perceived as technically superior, and further, due to complicated histories of science, colonialism, and postcolonialism, could have their ideas received more favorably [68].

## Methods

Three forms of data collection were used in an iterative approach – in-depth interviews, document review, and non-participant observation.

### In-depth interviews

Two forms of purposive sampling were utilized to select respondents for this study – maximum variation and snowball sampling [69]. Maximum variation sampling, an approach meant to capture similarities and differences across a diverse pool of stakeholders [69], enabled the selection of information-rich respondents representing each of the broad stakeholder groups, for example domestic, diasporic and foreign emergency physicians, government officials, medical college leadership and representatives from other new medical specialties (Table 1). Sampling decisions were taken by VS and SB, with input from RB. Data collection was conducted by VS and took place from March 2015 to March 2016, with the majority of interviews taking place in-person in India, in 11 cities/towns. A total of 87 interviews were conducted with 76 respondents, with 72 interviews taking place in-person, seven over the phone, and eight via Skype. 64 interviews were audio-recorded, and handwritten notes were taken during the interviews. Verbal consent was obtained from all respondents. Interviews were transcribed verbatim by a contracted transcriber, and then de-identified by the first author. Respondents, institutions and locations were masked using unique identifiers.

**Table 1:** Number and categorization of in-depth interview participants

Organizational categorization	Number of respondents
Current and former central government officials	3
Current and former regulatory institutions officials	12
Development partners officials	2
Domestic emergency medicine professionals	33
Diasporic and foreign emergency medicine professionals	14
Medical college leadership	6
Other new medical specialties stakeholders	5
Media representatives	1
Total	76

### Document review

We aimed to capture documentary evidence on a range of stakeholder categories – domestic emergency physicians, diasporic and foreign emergency physicians, government, medical colleges and other new medical specialties. VS identified 248 documents through a combination of internet searching and snowball sampling with respondents. Document categories included meeting minutes of key stakeholders, policy documents, correspondence between organizations, conference reports and brochures, and articles from Indian newspapers and magazines. VS conducted the review, and analyzed these documents for their relevance to the development of emergency medicine from the early 1990s until 2015.

### Observation

Maximum variation sampling and snowball sampling were utilized to purposefully select settings for observation [69]. VS and SB took these sampling decisions, aiming to select sites that reflected the diversity of the stakeholder groups involved in this case. VS observed six meetings – three national-level EM conferences and two ‘high-level’ expert meetings on topics related to EM (representing two different facets of the EM stakeholder network), and one state-level conference on health systems. Organizers of these conferences provided permission to observe these meetings. Data was collected in the form of extensive handwritten notes, which were later summarized as memos.

### Analysis

A version of the ‘framework’ method was utilized, a common analytic approach in policy research [70, 71]. The coding approach combined inductive and deductive approaches [70]. First, VS and SB developed a set of codes based on the conceptual framework, and then built on this list by reviewing memos generated from the interviews, observations and select documents to prepare an initial list of codes. Then, VS and SB, conducted line-by-line coding on six transcripts, from which codes were inductively generated [72]. VS and SB applied the new codebook to an additional seven transcripts, and based on this process, further condensed the codes into a final list through peer discussion.

Next, VS applied this final codebook to an additional 33 transcripts that were selected for in-depth coding due to the richness of the data presented in those interviews. VS and SB developed rich descriptions of the agenda setting, policy formulation and implementation stages of the case, using coded data and select documents. Coded data were reviewed, from which themes were developed pertaining to each of the policy phases. These themes were entered into a role-ordered matrix [73]. The remaining 41 interviews, relevant documents identified from the case study database, and observation data were reviewed to confirm or disconfirm themes, and present new information wherever possible. VS conducted respondent validation with three key informants by discussing key findings of the overall study and incorporating their feedback into the analysis [74].

In order to explore the role of power in the case, VS and SB began by attempting to link study data to existing theory, frameworks and concepts regarding power [54, 75]. VS and SB then inductively developed a conceptual framework based on this exploratory phase, and VS deductively applied this conceptual framework back to the full set of interviews, specifically by linking categories in the conceptual framework to existing codes in our codebook [76]. VS also drew upon selected documents and observations to triangulate these findings. VS and SB developed the analysis, and RB and AG provided input at multiple points in the process.

For this paper, we selected two aspects of that framework – socialization and legitimation – with an emphasis on the role of stakeholders from high-income countries, particularly the Indian diaspora.

## Results

**Fig. 1:**
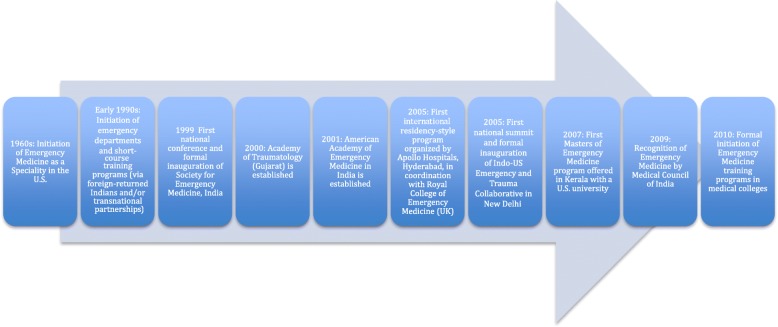
Key milestones in the development of emergency medicine in India

We present our findings chronologically, anchored by the milestones described in Fig. 1. We discuss stakeholders as part of one of three groups – domestic (stakeholders based in India), diasporic (stakeholders of Indian origin living outside of India, typically in a high-income setting), and foreign (stakeholders of non-Indian origin living outside of India, typically in a high-income setting). Table 2 describes the networks and relationships across these three groups. For reporting purposes, respondents are identified in the text by a unique identification code beginning with A (i.e., A1, A2, A3).

**Table 2:** Transnational networks in the development of emergency medicine in India

Professional association/group	Domestic members and partners	Diasporic and foreign members and partners
Society of Emergency Medicine, India	- Private hospitals - Private medical colleges	- American Association for Emergency Medicine in India - Royal College of Emergency Physicians - International Federation of Emergency Medicine - Individual contributors from high-income countries such as the U.S., U.K., Australia, Singapore, Saudi Arabia, etc.
Indo-US Emergency and Trauma Collaborative	- Public medical colleges - Private medical colleges	- U.S. medical colleges - British Association of Physicians of Indian Origin - U.S. Centers for Disease Control and Prevention - Individual contributors primarily from the U.S. and U.K
Academy of Traumatology	- Public medical colleges - Private hospitals	- Diasporic emergency physicians and other medical professionals from the U.S. - American Association for Physicians of Indian Origin
Other transnational partnerships	- Public medical colleges - Private medical colleges - Private hospitals	- Diasporic associations (for example, Andhra Pradesh Medical Graduates in USA) - Medical institutions and individual contributors from the U.S., U.K., Australia and other high-income settings

### Early development of emergency medicine in India through socialization

The development of emergency medicine as a medical specialty in India emerged out of a need to improve weak systems of emergency care in both public and private sectors, systems marked by poor quality, limited coordination, and inadequate prioritization by administrators and policymakers [77]. In high-income countries, starting with the US in the 1960s, emergency medicine also emerged as a solution to similar challenges [78]. As emergency medicine gained momentum in these settings, some examples of diffusion to India appeared in the early 1990s in both public and private sectors. The form of emergency medicine that took root in India was the ‘Anglo-American’ model, where clinical care was provided primarily in hospitals, rather than the ‘Franco-German’ model, which emphasized pre-hospital clinical care [79].

The pervasiveness and institutionalization of emergency medicine in many high-income countries gave the field considerable credibility with Indian stakeholders. Indian professionals who first became familiar with emergency medicine during their training in high-income countries, strongly believed that the specialty could positively impact the delivery of emergency care in India [A24, A29, A40, A39, A46, A91, A92, A93]. Emergency medicine stakeholders and policymakers stressed that a key parameter for introducing the specialty was its presence abroad, signaling a desire to keep pace with other countries [A12, A28, A71, A77].

*“Also, many countries were getting Emergency Medicine. There was an international effect. If Singapore was having Emergency Medicine, why not India too?”* Indian public sector medical college stakeholder.

*“I think basically it is, because rest is doing it, we are doing it.”* Indian public sector medical college stakeholder.

On the public sector front, in 1992, a proposal to establish the first formal training program for emergency medicine in the country was initiated at the All-India Institute of Medical Sciences (AIIMS) in New Delhi, the apex public institution for research, teaching and innovation in medical education in the country. However, due to disagreements within the AIIMS leadership about the need for emergency medicine, the program was ultimately only established twenty years later in 2012.

In the western state of Gujarat, the Bhuj earthquake of 2001 spurred some public sector medical college leaders in that state to actively explore options for improving emergency care in the state. Stakeholders travelled to the U.S. and gained exposure to emergency medicine, as noted by this respondent.

*“…and then they showed me the emergency department. Then I was surprised that this emergency department was not only tackling the trauma patients but they were also taken in the medical emergency also. So here is the department which deals with daily emergency. Whether it is medical or trauma, so medical, surgical, both emergencies. So that was the first time I thought that why we cannot do the same thing in India?”* Indian public sector medical college stakeholder.

Domestic stakeholders in Gujarat, working under the umbrella of the Academy of Traumatology, and in collaboration with U.S.-based Gujarati stakeholders and representatives of the American Association for Physicians of Indian Origin, began to advance system-wide emergency care reform in the public sector, including pre-hospital emergency care, short-course training programs in hospital-based care and later, postgraduate training in emergency medicine [A91, A92, A93]. Some respondents also discussed the relatively cohesive nature of the partnerships in Gujarat [A18, A92, A93], and the financial support to these initiatives provided by diasporic stakeholders [A93].

Private sector interest emerged in both hospitals and medical colleges. From the early 1990s, Apollo Hospitals took an active interest in emergency medicine, driven in large part by the exposure of its leadership to emergency care systems in the U.S. Apollo began to make connections with other interested medical professionals in India, and authorized the establishment of formal Emergency Department services in the Apollo system in the mid-1990s. Sporadic efforts to establish Emergency Departments in other private hospitals also began in the early 1990s. The financial resources of Apollo and other for-profit hospitals appears to have facilitated closer relationships with diasporic and foreign stakeholders, as for-profit hospitals were more easily able to fund travel and establish training courses, and also members of the diaspora [A17, A24, A42, A93].

*“Corporates were quite happy to employ people from abroad, coming into my arms saying, I want to provide a service.”* Diasporic stakeholder.

Some respondents described a key motivating factor behind corporate interest as being ‘brand building’ or marketing the advanced level of care in their facilities for the purposes of revenue generation [94] [A3, A53, A56, A93].

Private medical colleges were also early adopters of emergency medicine in India. Facilitated by exposure of leaders and other staff to emergency medicine in high-income countries such as the U.S., U.K. and Australia, colleges in the southern states of Karnataka and Tamil Nadu (such as Christian Medical College (Vellore), St. Johns Medical College and Sri Ramachandra Medical College in Chennai, Tamil Nadu) began establishing Emergency Departments and short-course training programs from 1994 onwards.

### The legitimation of emergency medicine through transnational professional associations and partnerships

In 1999, the first national association for emergency medicine, the Society for Emergency Medicine, India (SEMI), was formed with initial support from Apollo. The association pursued formal recognition for the field with regulators, established training programs, and organized conferences to raise awareness about the field. These efforts were strongly supported by members of U.S.-based Indian diaspora, who had organized themselves into an association called the American Association for Emergency Medicine in India (AAEMI). These stakeholders often built on the idea of India keeping up with more advanced countries, and drew upon their technical expertise to lend the idea legitimacy and credibility.

*“Just trying to keep pace with other developed countries that have the specialty recognized will also be important. If you’ve got international organizations they are saying, hey we have got this, and you as the country trying to keep pace with them in terms of the medicine and medical care provided, I think that would be important as well.”* Diasporic stakeholder.

Diasporic stakeholders, including recent emigrants and first- and second-generation citizens of high-income countries, were particularly motivated to promote emergency medicine in India, driven by their ‘vested interests’, ‘feeling’, or loyalty to the country, and through their desire to strengthen health care in India [A3, A4, A17, A18, A21, A22, A25, A26].

*“We expats or those of Indian origin have a vested interest in seeing India prosper and seeing patients get good care. This is where our families are from, and many of us have families here still.”* Diasporic stakeholder.

Some diasporic stakeholders felt that their value stemmed from a combination of their desire to improve Indian health care, their cultural familiarity with India, and their training and experience with emergency medicine in high-income countries. Some diasporic stakeholders, particularly those who had emigrated following medical training, also drew upon their deep knowledge of ground realities in India and their strong educational or regional networks in India to channel their efforts.

*“I don’t talk like, the American system is the best, you need to do this the US way, otherwise it’s not good. I know what is practicable in India, I tell them look, this is how we do in US, this is how it was done in England and this is how we used to do in India. We can modify it and combine it to a way it will work in India…”* Diasporic stakeholder.

AAEMI and other members of the diaspora from the U.S., U.K., Australia and Singapore participated actively in SEMI’s annual conferences and helped organize short- and long-term training programs. Foreign and diasporic stakeholders had the advantageous position of transferring knowledge regarding emergency medecine, particularly in the 1990s and early 2000s when the field was first developing in India. The diaspora played a particularly dominant role, serving as a bridge between their adopted countries and their country of origin [A3, A18, A22].

*“So these non-resident Indians they used to come and they used to tell that if it is happening in the country which have I worked why cannot it happen to my own home country. They play a big role into this. So they used to take people abroad, give them, show them the system, organize the annual conferences come in between and lot of handholding they did. And they got lot of Americans and British into this.”* Indian private sector stakeholder.

Domestic stakeholders reported gaining considerable knowledge, skills and expertise from foreign and diasporic stakeholders [A3, A4, A54, A23, A24, A28, AA124, A39, A93, A92]. The technical expertise from these stakeholders was perceived as superior given their longer experience with the discipline.

*“…if an expert from outside comes and tells you, it makes a huge difference, rather than your own people.”* Indian private sector stakeholder.

Supplementing this perception was the sense from diasporic and foreign stakeholders themselves that they possessed a body of knowledge reflecting ‘true’ principles of emergency medecine, therefore giving their activities in India more credibility [A18, A22, A29, A47].

Beginning in the late 1990s, organizations and individuals in the network actively pursued the recognition of emergency medicine as a medical specialty by the primary regulator in the country, the Medical Council of India. During a period of inaction from regulators in the 2000s, domestic, diasporic and foreign stakeholders, particularly from SEMI and AAEMI, decided that other options for postgraduate training needed to be pursued. The system of postgraduate medical education in India is unique in the availability of multiple uncoordinated pathways for specialist training, including medical colleges and private hospitals [80], and the presence of unregulated postgraduate training programs in the private sector (although the legality of such programs are increasingly being questioned) [48, 81]. These plans were also influenced by a growing demand for emergency medicine training programs from medical students and young doctors, spurred by increasing employment opportunities for Indian doctors to work in Emergency Departments in high-income countries such as the U.K, the Middle East and Australia.

Programs were initiated between some foreign and diasporic stakeholders and domestic partners, and these programs placed a strong emphasis on these stakeholders providing technical expertise [A3, A4, A17, A18, A25, A47, A62, A91]. Most notably, diasporic stakeholders appeared to have parlayed their roles within transnational networks into institutional partnerships between their home institutions and medical institutions in India, brokering relationships between diasporic and foreign individuals. Such partnerships were seemingly advantageous for all groups involved. In addition to reputational gains, foreign and diasporic stakeholders also found several international residency opportunities in India for U.S. residents, and domestic stakeholders gained technical expertise and a boost to their institutional branding.

*“A private hospital getting a ‘X U.S. Institution’ stamp on them, or a ‘Y U.S. Institution’ stamp on them in India, it’s a business model for them. They say, wow, we are getting marketing out of it. We can tell our patients that we get our education from this institution.”* Diasporic stakeholder.

However, as the 2000s progressed, domestic, diasporic and foreign stakeholders disagreed about these unregulated programs in the private sector [A4, A5, A19, A24, A42, A47, A88, A112, Documents]. Some domestic, diasporic and foreign stakeholders engaged with SEMI and AAEMI argued that given the scarcity of human resources, and the slowness of regulators in providing formal recognition, any type of training, even if unregulated, should help fill this demand. Beyond the issue of regulated courses, stakeholders also disagreed about whether the specialty was being sufficiently adapted to the Indian context.

*“There are two schools of thought in India. One school of thought says that India is for Indians alright, and if you bring a program to India from another country, it will never work in India, it has to address the needs of Indians. There is another school of thought that says that the mecca, the best quality of emergency care is in the USA and if the USA says it must be right, how can they be wrong.”* Diasporic stakeholder.

Domestic, and some diasporic, stakeholders noted that they felt that a trend of ‘Americanization’ was occurring with emergency medicine in India, suggesting an outsize presence of U.S. stakeholders in conferences and training programs. A few respondents also commented upon the underlying power differentials between diasporic and foreign stakeholders, noting the perception that some non-Indian stakeholders were sometimes given priority in national meetings during the early development of the specialty [A22, A29].

*“So there are many who resented the so called Americanization of emergency medicine for India and the Americans coming in to organize a program in India. Of course the bulk of Americans coming in were those of Indian origin but there were some who were not of Indian origin, of US origin who were doing it and they were given the prominence in many of these meetings. So you see, resentment was developing and so politics ruled SEMI for quite a few years. It was sad because that has slowed down the development of emergency medicine in India…”* Diasporic stakeholder.

These tensions bubbled over at various points in the development of the specialty in India. The first major fracturing took place in 2005 with the formation of another professional group, the Indo-US Emergency and Trauma Collaborative [82], a partnership comprised of public and private medical colleges and diasporic and foreign stakeholders, primarily from the U.S. This group decided to focus primarily on medical college training as a conduit for specialist training. INDUS-EM also leveraged the bureaucratic power of Indian public sector institutions such as AIIMS to advance policy objectives [A3, A19, A21].

Regardless of their membership with SEMI or INDUS-EM, almost all domestic stakeholders believed that postgraduate training was of paramount importance to building the specialty, and many believed that formal recognition from Medical Council of India, which would allow residency training programs to begin in medical colleges, was an essential policy objective. This was amplified by foreign and diasporic stakeholders who were also keen to see residency programs, similar to those offered in high-income countries, initiated in India [A5, A19, A66, A88].

*“So our goal was to encourage them to make it become an identified specialty to start specific residency programs in emergency medicine and to help pass on any lessons we have learnt over the 35 years of developing it in the United States.”* Foreign stakeholder.

Beyond the need for recognition from the Medical Council of India, it became evident that there were differences of opinion among stakeholders regarding the long-term trajectory for emergency medicine in India. For example, some diasporic and foreign stakeholders were more focused on clinical care, and took the view that engaging around issues of health equity and health systems strengthening were long-term goals.

*“It’s mostly academic. The [health] system as a whole was not really discussed much.”* Diasporic stakeholder.

### Progress for the specialty amidst growing fragmentation

The Medical Council of India formally recognized emergency medicine as a medical specialty in 2009. By the early 2010s, foreign and diasporic stakeholders were deeply involved in many aspects of emergency medicine in India – from sitting on formal committees at the national-level, to collaborating on training programs in private hospitals and medical colleges, to developing curricula. The level of engagement of these stakeholders ranged from offering postgraduate degrees through partnerships with Indian institutions to providing periodic inputs on curricula and department operations. There were numerous examples of domestic stakeholders drawing upon professional networks that were formed during their work experience in the U.S., U.K. and other high-income countries to access guidance on the functioning of Emergency Departments, develop curricula and improve other facets of training programs [A3, A48, A54, A91, A92].

*“So it took 4-5 years before even we realized that how much we had to grow and how many things we had to do, how important it was to learn things from other countries and other departments. And so we used to look at the curriculum of many other US universities, UK, what UK had done for their [Accident & Emergency]. What are the important things [that] need to be learned?”* Private sector medical college.

From the early 2010s, the tension between SEMI and AAEMI also seemed to be causing fissures in their partnership. Many respondents also likened the relationship between diasporic and foreign stakeholders on one hand, and domestic stakeholders on the other, to that of a parent and a child, one of both dependency and resistance.

*“…I retrospectively see that is natural. Why would you go as an international organization into another country? And start calling the shots. Till those people grow… when your child has grown they want to move out of the house and if you see, that is a very natural phenomenon happening. So, the international support came and did a lot of good. They organized them, got them to a level and then they were not liked because their own show was being stolen.”* Diasporic stakeholder.

Respondents commented on seemingly conflicting concepts of altruism and self-interest guiding diasporic and foreign stakeholders. These stakeholders often spoke of their involvement in altruistic terms, a sentiment echoed by some domestic stakeholders.

*“…this is what I take pride also in this, we are so proud of them, the US counterparts, that they give selfless service. That is what I say, true selfless service. You know without asking anything. So…they invested their time and resources in the things that has happened in India.”* Indian public sector stakeholder.

*“There are many altruistic people who were involved. Mostly the United States at first, but now also from Australia, UK and other western countries.”* Diasporic stakeholder.

However, several domestic stakeholders, and some diasporic stakeholders, were increasingly questioning this sense of altruism, perceiving that some self-interest, for example in the form of reputational or financial gain could also be underlying motivating factors [A4, A5, A17, A19, A21].

*“People come from outside, run their own systems. There are many universities in America and even the [X Institution in the U.K.] come to India and they run their own sweatshops in various specialties, they award their own degrees. I have never seen an Indian University coming to America or UK and giving degrees to its citizens. But because our country’s system is so open and broad these guys can venture out into Indian soil and start distributing the diploma so that is colonization of academics according to me and it is going on very actively in India right now.”* Diasporic stakeholder.

The conflict between SEMI and AAEMI played out in an intentionally reduced role for diasporic and foreign stakeholders at certain conferences, such as SEMI’s 2015 annual conference [Observation data, A4, A66]. Another consequence of the divide between the professional societies were divergent transnational affiliations. For example, stakeholders from INDUS-EM were less likely to participate in meetings or discussions with the International Federation of Emergency Medicine, or the American College of Emergency Physicians. Similarly, stakeholders from SEMI have not been engaged in discussions with INDUS-EM collaborators such as the Centers for Disease Control and the World Health Organization around strengthening emergency care in India.

By 2015, emergency medicine as a field of medical specialization was gaining momentum, despite the fragmentations and fluctuations within the stakeholder community. Respondents often explained that SEMI and AAEMI largely worked with the private sector, while INDUS-EM worked with the public sector. However in practice, there were notable exceptions to this, such as the involvement of AAEMI members in the development of an emergency medicine training program in an elite public sector medical college, the initiation of a relationship between INDUS-EM and the National Board of Examinations (a regulatory agency administering formal training programs in the private sector), and the involvement of private medical colleges with INDUS-EM. Further, in this time period, some partnerships between Indian and high-income country stakeholders were maintained, while others waned. One major consequence of this fragmentation was the diversity in resulting training programs and partnerships, with few opportunities for standardization of curricula and protocols. Another consequence is that SEMI and INDUS-EM were engaged in parallel policy efforts, such as the introduction of national emergency care legislation modeled after emergency care legislation in the U.S. Finally, both groups were taking tentative steps in positioning India as a conduit to establish and influence emergency medicine in other South Asian countries, such as Sri Lanka (Observation).

## Discussion

This analysis of the role of high-income country stakeholders in the development of emergency medicine through the lens of socialization and legitimation reveals the underlying dynamics that have fundamentally shaped the growth of the field in India. Our analysis suggests that both the socialization of domestic stakeholders to emergency medicine in high-income settings, and the active involvement of diasporic and foreign stakeholders in promoting emergency medicine within India may help explain its acceleration in that context, particularly when compared to other new medical specialties, such as palliative medicine and infectious diseases, that emerged around the same time but had more limited involvement from high-income country stakeholders [83]. Many in the medical community saw emergency medicine as an important solution to the serious challenges of emergency care in India. Yet, the focus in this time period was on emergency medicine, inadvertently prioritizing a medicalized form of tertiary care, rather than emergency care more broadly.

The socialization of domestic stakeholders to emergency medicine directly flows from a long history of LMIC stakeholders adopting and adapting ideas from undercurrents of colonial and postcolonial histories, globalization, and innovations in communication and technology [84], and reinforced by a latent hegemony in the ideas emanating from those countries [58]. This socialization is also reinforced through the 'imagined community of clinicians' practicing global biomedicine, through which domestic, diasporic and foreign physicians are networked and connected [84]. As a result, socialization on the part of domestic stakeholders, combined with active legitimation by diasporic and foreign stakeholders, may further a tendency in India and other LMICs to sometimes adopt policies without adequate reflection on their contextual appropriateness and effectiveness [84]. As noted by Zachariah [16], “the unreflective transfer of knowledge developed in a Western population and for the Western health system to the Indian setting has led to a mismatch between the structure of the health problem and the knowledge that is being used to address it.” 

Several studies on the engagement of high-income country stakeholders in development agendas within LMICs similarly suggest deep power asymmetries with national stakeholders, and a few also suggest a tenuous line between altruism and self-interest in the motivations of foreign and diasporic stakeholders [85–87]. Our study contributes to a growing understanding of the influence of players beyond ‘typical’ external stakeholders, such as international health organizations and donors, in developing health policy. In a rapidly globalizing world, the diaspora, multinational companies, and consortia of medical professionals and medical institutions have a growing influence on policy trajectories. Building on existing asymmetries, power further manifested in this case through the exportation of ‘valuable’ knowledge by the foreign and diasporic stakeholders. Such trends are not new. Regarding a 1985 initiative by the Medical Council of India and the American Association of Physicians of Indian Origin to train doctors in India on advancements in medical technology, the Economic and Political Weekly presciently warned, that the initiative “will promote and encourage a value system in which the ‘best’ medicine becomes synonymous with high- tech medicine” [35]. These concerns seem relevant three decades later, even more so given the increasing globalization of biomedicine. The socialization and legitimation of models from high-income settings in India is also noteworthy as Indian stakeholders seemed less enthused by acquiring knowledge from other LMICs; conversely, some Indian stakeholders were positioning India as a conduit of emergency medicine to other LMICs.

Our study finds that stakeholders from high-income countries, particularly the Indian diaspora, effectively used their role as technical ‘ambassadors’ to actively legitimate emergency medicine in India. Further, foreign and diasporic actors form national and regional-level transnational networks that allow them to parlay their network connections into partnerships between their home institutions and institutions in India, an example of the multi-dimensional nature of transnational flows [45]. The technical expertise of foreign and diasporic stakeholders was buttressed by other forms of power – financial power, network power and bureaucratic power – from both within and outside India, creating the conditions for successfully legitimizing the field. For example, the financial power of corporate for-profit hospitals facilitated a platform for diasporic and foreign stakeholders to establish emergency medicine initiatives, the bureaucratic power of AIIMS provided a lift to the transnational INDUS-EM group in their efforts to gain formal recognition for the specialty, and the network and financial power of the Gujarati diaspora in the U.S. facilitated efforts to improve emergency care in Gujarat. In this way, the transfer of knowledge and ideas in India cannot be seen as an apolitical exercise; rather, power strongly modulates the type of knowledge that gains traction, and therefore impacts any eventual modifications to the health system that emerge as a result.

Our findings suggest that the transnational networks engaged with transferring medical knowledge are heterogeneous, forging multiple and sometimes contested partnerships with divergent goals. The strength of these partnerships also appears to depend on the scale at which they are formed. For example, national partnerships in this case appeared more susceptible to conflict and dissolution, while regional partnerships and networks appeared more cohesive, as in the case of Gujarat. The strength of such diasporic networks from certain regions of India, such as Gujarat, has also been observed in other sectors, such as politics and culture [33]. Further, the pluralistic nature of the diaspora – recent emigrants, first-, second- and third-generation – in this case added diversity in terms of ideologies, philosophies and objectives. In the absence of a structured system for coordinating medical specialties within India, this heterogeneity in transnational partnerships becomes relevant. For example, as existing governance structures do not enable the standardization of curricula for medical specialties in India, these myriad transnational partnerships introduced different curricula or protocols for these specialties, surfacing new ideas and innovations, but exacerbating the intractable lack of coordination and standardization for health services in India [88].

The role of the Indian diaspora as brokering the transfer of biomedical knowledge and ideas also warrants further attention. The involvement of the diaspora in development is viewed by many in India as a positive development [29]. Their engagement has been strongly supported by state and central government authorities in India [37, 42], and in recent years, is increasingly seen as a major contributor to India’s pro-business economic agenda [43]. However, the involvement of the diaspora in biomedical knowledge transfer has largely occurred without necessary critical analyses of its intended and unintended consequences [45]. Due to the emphasis on tertiary care in the Indian health sector over several decades, there is a favorable market for the transfer of medical knowledge, most notably in the context of corporate hospitals [38]. However, the the backlash observed in this case from some Indian stakeholders, and the negative impact of this backlash on formerly productive collaborations, complicates the narrative in some development literature of positive, unidirectional knowledge flows [29]. We posit that for many diasporic stakeholders, a combination of their clinical training and experience in tertiary medical institutions in high-income countries, the relationships cultivated with and by elite, private sector Indian stakeholders, and the broader context of pro-business diasporic connections promoted by the state, might de-emphasize a focus on rural health care and vulnerable populations.

### Limitations

This study presented several limitations. First, our study is a single case of one new medical specialty in one country; multiple case studies present further opportunities for comparison and are therefore considered more analytically robust [89]. Second, while we attempted to capture a comprehensive and diverse range of viewpoints, we were unable to interview all stakeholders involved in the development of emergency medicine in India; as a result, our findings might not capture certain perspectives. We addressed this limitation by triangulating data sources and through member checking. Third, due to the sensitive nature of questions around power, we were often unable to explicitly engage in dialogue around power with our respondents, and therefore, the analysis relied almost exclusively on our interpretation of the data, and therefore could potentially reflect our biases. We attempted to address this limitation through member checking by discussing certain findings with key respondents, and peer debriefing through frequent discussion amongst the co-authors.

## Conclusion

The transfer of medical knowledge and ideas from high-income countries to India has been portrayed as a largely positive phenomenon. Using the case study of the development of emergency medicine as a medical specialty, this analysis of power uncovers a complex picture of the role of stakeholders from high-income countries, particularly the Indian diaspora, in the transfer of medical knowledge to India. Domestic stakeholders were socialized to concepts of emergency medicine through work experience in high-income countries, facilitating the development of the specialty in Indian hospitals upon their resettlement in India. Foreign and diasporic stakeholders actively promoted the field through transnational networks, conferences, and institutional training partnerships. These activities have seemingly played a critical role over the last few decades, leading to increased availability and quality of emergency care, particularly in hospitals in urban and peri-urban parts of the country. However, the nature of their involvement was also characterized by a fragmented landscape of professional groupings and postgraduate training programs, the prioritization of specialist programs over health systems approaches, and a perceived lack of adaptation of emergency medicine as practiced in high-income countries to Indian realities. More research, particularly analyses of power, are required to explore the transfer of other forms of medical knowledge, such as other medical specialties, models of clinical care, and medical technologies, from high-income countries to India. Such research will help us understand how and why certain forms of biomedical care are privileged in India and whether additional efforts are required to align such efforts with broader challenges of health systems strengthening and health equity.

## Abbreviations

AAEMI: American Association for Emergency Medicine in India
AIIMS: All-India Institute of Medical Sciences
EM: Emergency medicine
INDUS-EM: Indo-US Emergency and Trauma Collaborative
SEMI: Society for Emergency Medicine, India

## Footnotes

1 Although not the focus of this paper, we recognize that the role of Indians transferring knowledge from high-income settings either through permanent resettlement in India or through diasporic ties is not limited to the private medical sector; for example, leading public health activists in India have been trained in high-income countries, and continue to engage actively in transnational networks, and diasporic networks such as Vellore Christian Medical College Foundation and Association for India’s Development are closely involved in public health and social justice activities in India.
